# Functional Characterization of a Rice Thioredoxin Protein OsTrxm and Its Cysteine Mutant Variant with Antifungal Activity

**DOI:** 10.3390/antiox8120598

**Published:** 2019-11-29

**Authors:** Seong-Cheol Park, Il Ryong Kim, Jin-Young Kim, Yongjae Lee, Su-Hyang Yoo, Ji Hyun Jung, Gang-Won Cheong, Sang Yeol Lee, Mi-Kyeong Jang, Jung Ro Lee

**Affiliations:** 1 Department of Polymer Science and Engineering, Sunchon National University, Suncheon 57922, Korea; schpark9@gnu.ac.kr (S.-C.P.); jyfrog@hanmail.net (J.-Y.K.); 2 Division of Ecological Safety Research, National Institute of Ecology, 1210 Geumgang-ro, Maseo-myeon, Seocheon 33657, Korea; kir6060@nie.re.kr (I.R.K.); hyang77@nie.re.kr (S.-H.Y.); 3 Division of Applied Life Science (BK21+ Program), Plant Molecular Biology and Biotechnology Research Center (PMBBRC), Gyeongsang National University, Jinju 52828, Korea; henia@korea.kr (J.H.J.); sylee@gnu.ac.kr (S.Y.L.); 4 Department of Nutrition and Food Science, Texas A&M University, College Station, TX 77843, USA; specbiotechllc@gmail.com; 5 Goseong Agricultural Development/Technology Center, Goseong 52930, Korea; 6 Division of Applied Life Sciences and Research Institute of Natural Science, Gyeongsang National University, Jinju 52828, Korea; gwcheong@gnu.ac.kr; 7 The Research Institute for Sanitation and Environment of Coastal Areas, Sunchon National University, Suncheon 57922, Korea

**Keywords:** OsTrxm, active cysteine, antifungal protein, rice, pathogen

## Abstract

Although there are many antimicrobial proteins in plants, they are not well-explored. Understanding the mechanism of action of plant antifungal proteins (AFPs) may help combat fungal infections that impact crop yields. In this study, we aimed to address this gap by screening *Oryza sativa* leaves to isolate novel AFPs. We identified a thioredoxin protein with antioxidant properties. Being ubiquitous, thioredoxins (Trxs) function in the redox balance of all living organisms. Sequencing by Edman degradation method revealed the AFP to be *O. sativa* Thioredoxin m-type isoform (OsTrxm). We purified the recombinant OsTrxm and its cysteine mutant proteins (OsTrxm C/S) in *Escherichia coli*. The recombinant OsTrxm proteins inhibited the growth of various pathogenic fungal cells. Interestingly, OsTrxm C/S mutant showed higher antifungal activity than OsTrxm. A growth inhibitory assay against various fungal pathogens and yeasts confirmed the pertinent role of cysteine residues. The OsTrxm protein variants penetrated the fungal cell wall and membrane, accumulated in the cells and generated reactive oxygen species. Although the role of OsTrxm in chloroplast development is known, its biochemical and molecular functions have not been elucidated. These findings suggest that in addition to redox regulation, OsTrxm also functions as an antimicrobial agent.

## 1. Introduction

As sessile organisms, plants are continuously exposed to diverse biotic/abiotic factors, such as pathogens, adverse climates, and hormones. In their defense against external pathogenic attacks, antimicrobial proteins play an important role. However, mechanisms of action of plant antifungal proteins (AFPs) are not well understood. This is significant since decreases in crop yield ire often attributed to fungal infections. In this study focusing on *Oryza sativa*, we isolated and characterized a thioredoxin protein, establishing it as a novel AFP.

Thioredoxin proteins act as antioxidants in various ubiquitous cellular processes, including the activation of ribonucleotide reductase, regulation of transcription factors, ROS scavenging, and the photosynthetic pathway in plant cells [1,2,3,4,5,6]. Trx proteins contain two active cysteine residues in the conserved redox disulfide/dithiol motif of “Trx-fold,” Trp–Cys–Gly (Pro)–Pro–Cys [7]. According to subcellular localization and sequence similarity of Trx proteins, 15 subgroups have been classified [8,9,10,11]. The Trx-m, -f, -x, -y, and -z are commonly detected in the chloroplast fractions, Trx-h is located in cytosol or nucleus, and Trx-o is found in mitochondria [12,13]. Trxm was originally identified as a regulator of photosynthetic function [14]. Recently, Trxm was suggested a function as a key regulator in photosynthetic redox-regulation [15,16].

Intriguingly, several Trx-like proteins, including Trx h1, h3, h4, and h5 in Arabidopsis, act as molecular chaperone [17]. The inhibitory activities of *Arabidopsis* Trx-h5 and rice TDX against pathogenic infection have also been reported [18,19]. Although it can be expected that isolated OsTrxm may function as a regulator of plant defense mechanisms, there is little information published on the biochemical defense mechanisms and an antimicrobial function of OsTrxm.

In this study, we identified OsTrxm to be a rice antifungal protein. We characterized the natural antimicrobial activity of OsTrxm and examined its potential mechanism as an antimicrobial agent under environmental stress conditions. To our knowledge, this is the first report of the antifungal property of OsTrxm as a rice antioxidant protein.

## 2. Materials and Methods

### 2.1. Materials

FNR-675 N-hydroxysuccinimide (NHS) ester was purchased from BioActs (Incheon, Korea). Superdex^TM^ 200 (10/300); CM sepharose^®^ Fast Flow and DEAE sepharose^®^ Fast Flow were bought from GE Healthcare (Piscataway, NJ, USA). The 2′,7′-dichlorofluorescein diacetate (DCF-DA) and MitoSOX Red were obtained from Molecular Probes Inc., (Eugene, OR, USA). Histatin 5 and melittin were synthesized by using a microwave peptide synthesizer (Discover Bio^TM^, CEM Co., Matthews, NC, USA) via solid phase method. All other chemicals were of reagent grade and all solvents were HPLC grade.

### 2.2. Fungal Cells

*Aspergillus flavus* (KCTC 6905), *A. fumigatus* (KCTC 6145), *Candida albicans* (KCTC 7270), *C. catenulate* (KCTC 7642), *C. tropicalis* (KCTC 7221), *Fusarium moniliforme* (KCTC 6149), *F. solani* (KCTC 6326), *Penicillium verrucosum* (KCTC 6265), *Phytophthora nicotianae* (KCTC 40164), *Trichoderma harzianum* (KCTC 6043), and *T. viride* (KCTC 6047), were purchased from the Korea Collection for Type Cultures (KCTC). Drug-resistant *C. albicans* (CCARM 14007) was obtained from the Culture Collection of Antimicrobial Resistant Microbes (CCARM, Seoul, South Korea). Molds were pre-cultured on potato dextrose agar (PDA) at 28 °C for 4 days and yeast cells were pre-grown in yeast extract peptone dextrose (YPD) broth at 28 °C for 24 h.

### 2.3. Purification of Antifungal Protein in Rice

The rice leaves rapidly frozen in liquid nitrogen were ground to a fine powder by using a mortar and pestle. The powders were extracted for 4 h at cold room in 25 mM Tris-HCl buffer, pH 7.4, containing 1.5 M LiCl and protease inhibitors (1 mM phenylmethylsulfonyl fluoride, 100 mM amino-*n*-caproic acid, 10 mM EDTA, and 5 mM benzamidine-HCl). After centrifugation at 15,000× *g* at 4 °C for 30 min, the supernatants were dialyzed in 25 mM 2-(*N*-morpholino)ethanesulfonic acid (MES, pH 5.5) containing 10 mM NaCl (cut-off of 1000 Da). AFP was purified by a cation and anion exchange chromatography and size exclusion chromatography (SEC) using a Bio-Rad BioLogic DuoFlow^TM^ System (Hercules, CA, USA). The dialyzed samples were passed directly in a CM sepharose cation-exchange column (4.6 × 10 cm) equilibrated in 25 mM MES, pH 5.5. The unbound fraction dialyzed with 25 mM Tris-HCl, pH 7.4 containing 10 mM NaCl (equilibration buffer), was applied to a DEAE sepharose anion-exchange column (4.6 × 10 cm). After washing column with three bed volumes of equilibration buffer, the bound proteins were eluted with a linear gradient of 0.01–1 M NaCl in 25 mM Tris-HCl, pH 7.4. The fractions with antifungal activity against *F. moniliforme* were pooled and concentrated by ultrafiltration. To obtain AFP with high quality, the concentrated protein solution was applied to a Superdex 200 gel filtration column equilibrated with phosphate buffered saline (PBS, pH 7.4) at 0.6 mL/min. The purity of proteins in fractions were confirmed on a 12% SDS-PAGE gel. The N-terminal amino acid sequence analysis using Edman degradation was conducted to identify the purified AFP. The protein isolated from rice were prepared by the pulsed liquid method using PVDF membrane (Applied Biosystems, Foster City, CA, USA). The PTH column for HPLC and Procise PC software 2.1 were used to detect peptides at 269 nm. The fast gradient was performed with two solvents, A3 (3.5% tetrahydrofuran in water) and B2 (12% isopropanol in acetonitrile), at a flow rate of 325 μL/min in a Procise 492 Protein Sequencing System (Applied Biosystems, Foster City, CA, USA).

### 2.4. Cloning of OsTrxm Variants and Expression in E. coli

OsTrxm (Os12g0188700) and the cysteine double mutant (C95S/C98S) OsTrxm (herein referred to as OsTrxm C/S) were generated from an *Oryza sativa* cDNA library using PCR. A DNA fragment of OsTrxm was amplified using the OsTrxm forward primer (5’-GAATTCATGGCGTTGGAGACGTG-3’) and OsTrxm reverse primer (5’-CTCGAGTCAGCTGCTGACGTAC-3’). The cysteine mutant OsTrxm C/S was generated by site-directed mutagenesis using overlap extension using PCR [20,21]. To obtain the two cysteine mutant gene, we additionally used OsTrxm5CS3 primer (5’-GTGGTCCGGACCGTCCAGGATG-3’) and OsTrxm3CS5 primer (5’-CATCCTGGACGGTCCGGACCAC-3’). Their DNA fragments were ligated into pET28(a) expression vector to purify recombinant proteins by his-tag. The transformants in BL21 (DE3) cells were cultured at 37 °C in LB medium and the variant proteins were induced by the addition of 0.4 mM isopropyl-ß-D-thiogalactopyranoside (IPTG). To isolate recombinant OsTrxm and OsTrxm C/S proteins, the extracted samples by sonication were applied to a Ni-NTA agarose (Qiagen, Chatsworth, TX) and they were eluted by a linear gradient of 10–250 mM imidazole, followed by further purification in FPLC System using a Superdex^TM^ 200 10/30 column with phosphate-buffered saline (PBS, pH 7.4). The purity of OsTrxm and OsTrxm C/S was determined using SDS-PAGE and the proteins were used to analyze their biochemical properties [22].

### 2.5. Antifungal Assay

The antifungal activity of the purified recombinant proteins was investigated by using a radial growth inhibition and microtiter plate assay. Potato dextrose agar (PDA) plates with pre-grown *T. harzianum*, *F. moniliforme,* and *P. verrucosum* fragments were incubated for 48 h at 28 °C until their mycelia were grown up to approximately 2–3 cm from the plate center. The samples on paper discs were placed at a distance of 1 cm from mycelia and the plates were incubated for an additional 72 h at 28 °C [23].

To investigate the 50% inhibitory concentration (IC_50_) against various filamentous fungi and yeasts, the conidia from five-day-old cultures were collected by treatment of 0.08% Triton X-100 on fungal mycelium. The conidia (final 10^4^ spore/mL) suspended in Potato dextrose (PD) (for filamentous fungi) or YPD (for yeast) medium was placed in 96-well microtiter plates where proteins were serially diluted. The hyphal growth inhibition of samples incubated at 28 °C for 24 to 48 h were observed by an inverted light microscope. The IC_50_ was evaluated to quantify antifungal effect by MTT assay. The IC_50_ values against individual fungus were presented as the lowest concentration to reduce the fungal germination or cell growth of yeast by more than 50%. All assays were performed three times [24].

### 2.6. Cellular Distributions of OsTrxm Protein Variants

The purified recombinant OsTrxm protein variants were incubated with FNR-675 NHS dye in PBS at a molar ratio of 1:1 for 2 h. To remove the unreacted fluorescent dye, samples were applied to a desalting column prepacked by Bio-Gel^®^ P-6DG Gel (Bio-Rad Laboratories, inc., Hercules, CA, USA) with PBS. FNR-675 labeled OsTrxm proteins of IC_50_ concentrations were added at the fungal cell suspensions (10^4^ conidia/mL) and incubated on a 24-well microtiter plate for 4 h at 28 °C. After washing with PBS buffer for three times, the fungal cells were spread onto poly-L-lysine-coated glass slides and the images were recorded by confocal laser scanning microscopy (CLSM, LSM 510 META, Gottingen, Germany). Furthermore, the cells incubated with FNR 675-labeled OsTrxm proteins of IC_50_ concentrations were analyzed by 5000 events/sample by reading through a 660/20 band-pass filter using FACS [25].

### 2.7. Measurement of Intracellular ROS Levels

To examine the oxidative damage in *C. albicans*, the cells were incubated with OsTrxm proteins and melittin at its IC_50_ concentration for 12 h. After incubation, the cells stained with 100 μM DCF-DA for 1 h, were observed by fluorescence microscope. Furthermore, in order to investigate extracellular ROS generation, the 96-well plates were analyzed by using a FOBI imaging system (Neoscience Co., Suwon-si, Gyeonggi-do, Korea) with a green fluorescent filter and the fluorescence intensity was represented as rainbow colors. The ROS-generating cells were measured by using flow cytometry at 5000 events/sample [24].

To investigate mitochondrial superoxide (SOX) in fungal cells, the MitoSOX Red probe was used. *A. flavus* conidia were treated with histatin 5, OsTrxm, and OsTrxm C/S at their IC_50_ concentrations for 8 h and were stained by following the manufacturer’s protocol for MitoSOX Red. The cells were digitally recorded by using a fluorescence microscope.

### 2.8. Scanning Electron Microscopy (SEM)

To investigate morphological alteration of *C. albicans*, each OsTrxm variant protein was incubated with precultured *C. albicans* cells (1 × 10^6^ cells/mL) at four-times IC_50_ value for 4 h. *C. albicans* cells treated with proteins were dropped on poly-L-Lysine coated glass slides and incubated for 30 min at room temperature (RT). The attached cells were carefully washed with 0.1 M HEPES buffer (pH 7.4) and fixed with 0.1 M HEPES buffer (pH 7.4) containing 2% glutaraldehyde (*v/v*) at RT. The fixed cells were sequentially dehydrated by graded ethanol (50%/75%/100%) and dried by evaporation of hexamethyldisilazane (HMDS). After sputter-coating by gold-palladium, the cell morphology was observed by field emission scanning electron microscopy (EF-SEM, JSM-7610F Plus; JEOL, Ltd., Tokyo, Japan) [24].

## 3. Results and Discussion

### 3.1. Isolation and Characterization of An Antifungal Protein from Rice Plants

**Figure 1:**
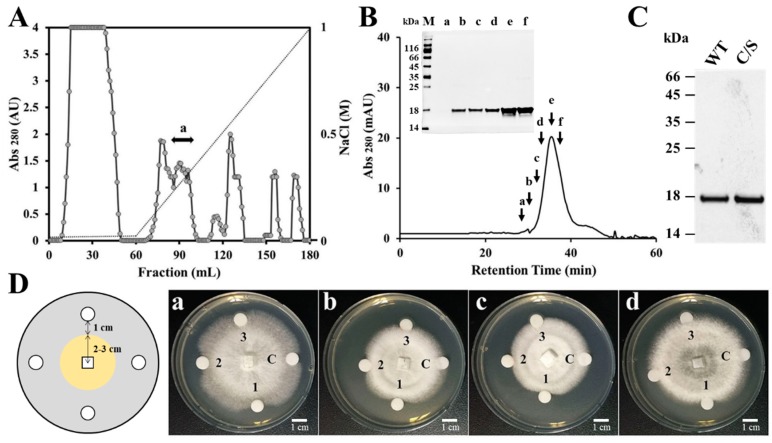
Isolation of a rice antifungal protein and growth inhibition of OsTrxm variant proteins. (**A**) The fraction unbounded in CM sepharose column was executed under DEAE sepharose column, which resulted in purification of OsTrxm in the indicated fraction (a). Dotted line presents a linear gradient of NaCl. (**B**) Fraction with antifungal activity was loaded on Superdex 200 column at 0.6 mL/min for 60 min and the indicated fractions were ascertained in a SDS-PAGE (inset figure) M: size marker; a–f: FPLC fractions. (**C**) Recombinant OsTrxm (WT) and OsTrxm C/S (C/S) proteins were purified by using Ni-NTA and Superdex 200 columns. (**D**) Growth inhibition of OsTrxm and its mutant protein in solid cultures of *Trichoderma harzianum* (a), *Fusarium moniliforme* (b), *Penicillium verrucosum* (c), and *T. viride* (d). C: control; 1: OsTrxm C/S; 2: histatin 5; 3: OsTrxm, a scheme of radial growth inhibition assay (left panel).

Previous results have suggested that the gene expression of plant antioxidant proteins is changed by various environmental factors, and that functions of these proteins are related to plant defense mechanisms [26,27,28,29]. To isolate a novel AFP from natural sources, crude proteins were extracted from the rice leaves which were homogenized with liquid nitrogen. Natural AFP was purified by using a combination of ion exchange chromatography and SEC. CM sepharose resin was applied to remove cationic proteins and the unbound proteins were next subjected to DEAE sepharose, followed by fractionation via a linear gradient of 0.01–1M NaCl (Figure 1A). We performed antifungal assay with the fractions after dialysis in PBS and the fractions with antifungal activity were pooled and concentrated. SEC was applied to purify pure AFP in Superdex 200 column (Figure 1B) and the antifungal effect of the presented fractions was ascertained against *F. moniliforme* conidia (data not shown). The isolated AFP was identified by N-terminal sequence via Edman degradation, resulting in a match to OsTrxm (XP_015620324.1) (data not shown). To investigate antifungal effects and mechanism of OsTrxm, the *OsTrxm* gene was cloned from a rice leaf cDNA library. Moreover, to investigate a role of cysteine residues in antifungal action, the cysteine double mutant, in which two cysteine residues were substituted to serine residues (double mutation of C95S and C98S), was generated from the cloned *OsTrxm* gene. The recombinant OsTrxm proteins were purified by using Ni-NTA resin and SEC (Figure 1C).

The antifungal activity of recombinant OsTrxm and OsTrxm C/S was indicated by a crescent-shaped zone of inhibition on the mycelia of *T. harzianum*, *F. moniliforme*, *P. verrucosum,* and *T. viride* (Figure 1D). Histatin 5, an antifungal peptide was used as a positive control. It potently inhibited the mycelial growths of four tested fungal cells when the cells were treated with 10 μg of the peptide. The inhibitory zone of OsTrxm C/S protein treated at 1.75 μg was similar to that obtained from the treatment of 10 μg of the histatin 5. In contrast, OsTrxm did not show inhibition at 7 μg, which was four times the concentration of the mutant OsTrxm C/S used in the treatment. Large amounts of proteins are used in solid culture conditions because it is difficult for the proteins to diffuse in agar. However, in liquid culture conditions, there is more potent antifungal activity due to the direct and multi-dimensional interactions with the fungal cell wall that can be witnessed.

**Figure 2:**
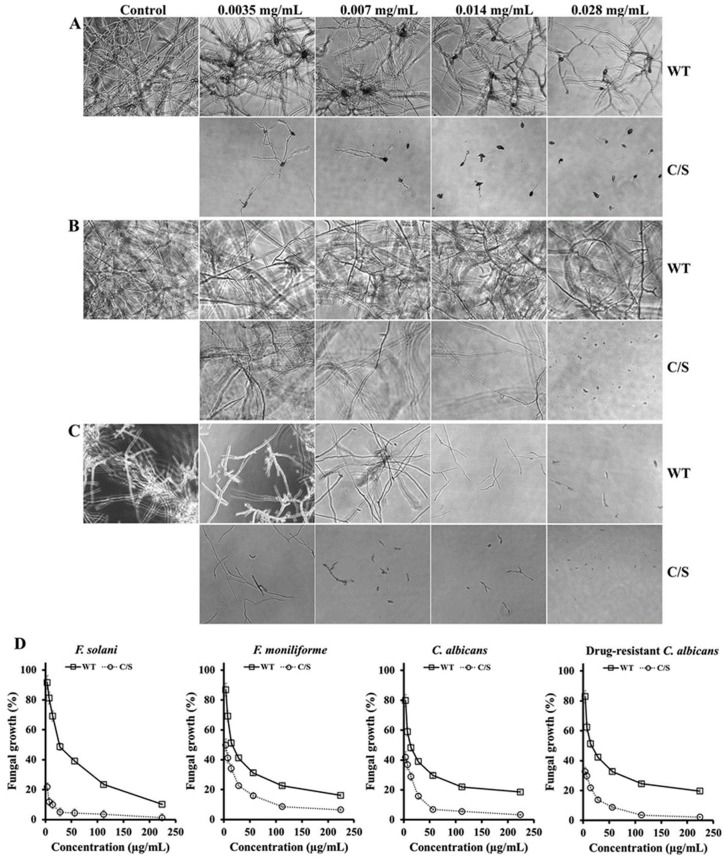
Antifungal effects of OsTrxm and its mutant protein in liquid culture. *Fusarium solani* (**A**), *T. viride* (**B**), and *F. moniliforme* (**C**) fungal cells were observed in the presence of OsTrxm (WT) and OsTrxm C/S (C/S) by using microscope. Magnification is 100×. (**D**) Dose-dependent growth inhibition of OsTrxm (WT) and OsTrxm C/S (C/S) was measured by MTT assay.

Furthermore, the antifungal activity of recombinant OsTrxm was evaluated in 12 strains belonging to eight filamentous fungi and four yeasts. Table 1 shows IC_50_ values of recombinant OsTrxm proteins against 12 strains (eight filamentous fungi and four yeasts), ranged from 7 to 28 μg/mL for inhibition of mold germination and yeast proliferation in liquid culture conditions. OsTrxm C/S protein showed a significant increase in the fungal growth inhibitory effect (Table 1). These results suggest that the structural change via mutation of the cysteine residues may enhance the antifungal action of OsTrxm. Figure 2 shows a potent antifungal action of OsTrxm proteins in the inhibition of fungal germination under liquid culture conditions. In Figure 2D, dose-dependent growth inhibition of OsTrxm proteins in *C. albicans, F. solani,* and *F. moniliforme* cells was also measured by MTT assay.

### 3.2. Cellular Localization of OsTrxm Proteins in Fungal Cells

**Figure 3:**
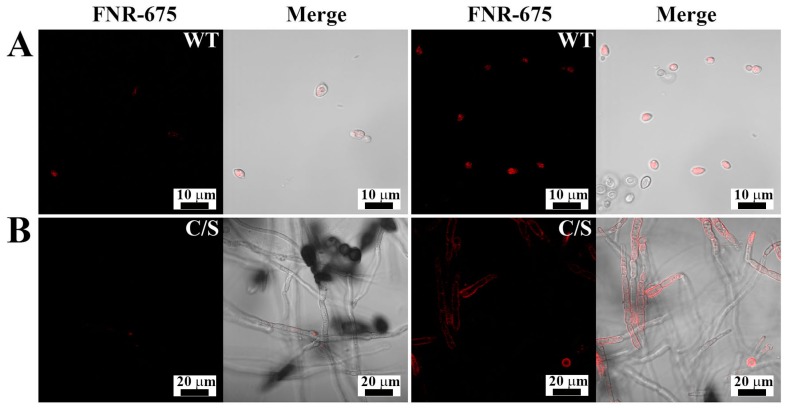
Cellular distributions of FNR-675-labeled OsTrxm proteins in *Candida albicans* (**A**) and *F. solani* (**B**) cells. The cells were treated with OsTrxm variants (WT: OsTrxm and C/S: OsTrxm C/S) at their IC_50_ concentration.

To determine the cellular distribution of OsTrxm and the C/S mutant, the FNR-675 fluorescent dye was conjugated to the primary amine group of a cationic amino acid in the protein by an amide bond’s formation. To minimize the artificial effects of protein, such as hydrophobicity, dye labeling to proteins was performed at molar ratio of 1:1. Figure 3 shows cellular distributions of FNR-675 labeled OsTrxm proteins in *C. albicans* and *F. solani* cells. The OsTrxm C/S mutant showed higher accumulation than OsTrxm in the cell walls and cytoplasms of treated cells. Although compositions of fungal cell walls are variable, fungal cells can transmit macromolecules into cytosol by endocytosis as well as other eukaryotic cells. We propose that intracellular penetration of OsTrxm proteins may be due to endocytosis.

### 3.3. Celluallar ROS and Mitochondrial SOX Generation

**Figure 4:**
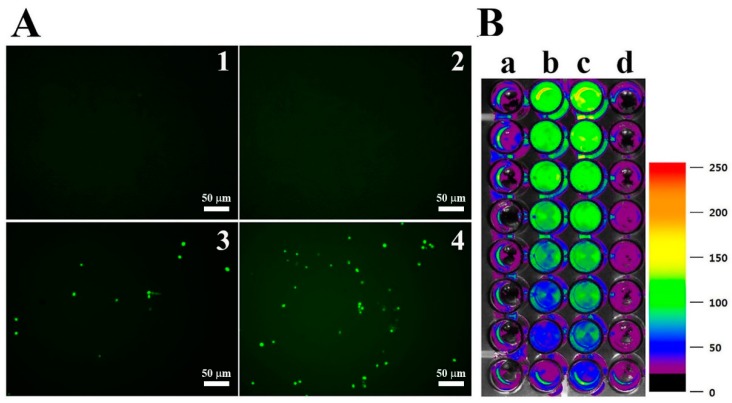
ROS generation by OsTrxm proteins in *C. albicans*. (**A**) Cells were treated with OsTrxm proteins at the IC_50_ concentration for 12 h, and they were stained with 0.5 μM DCF-DA for 30 min. 1: control; 2: melittin; 3: OsTrxm; 4: OsTrxm C/S. (**B**) Merged 96-well plate between bright image and fluorescent levels of DCF in *C. albicans* cells. a: control; b: OsTrxm; c: OsTrxm C/S; d: melittin.

**Figure 5:**
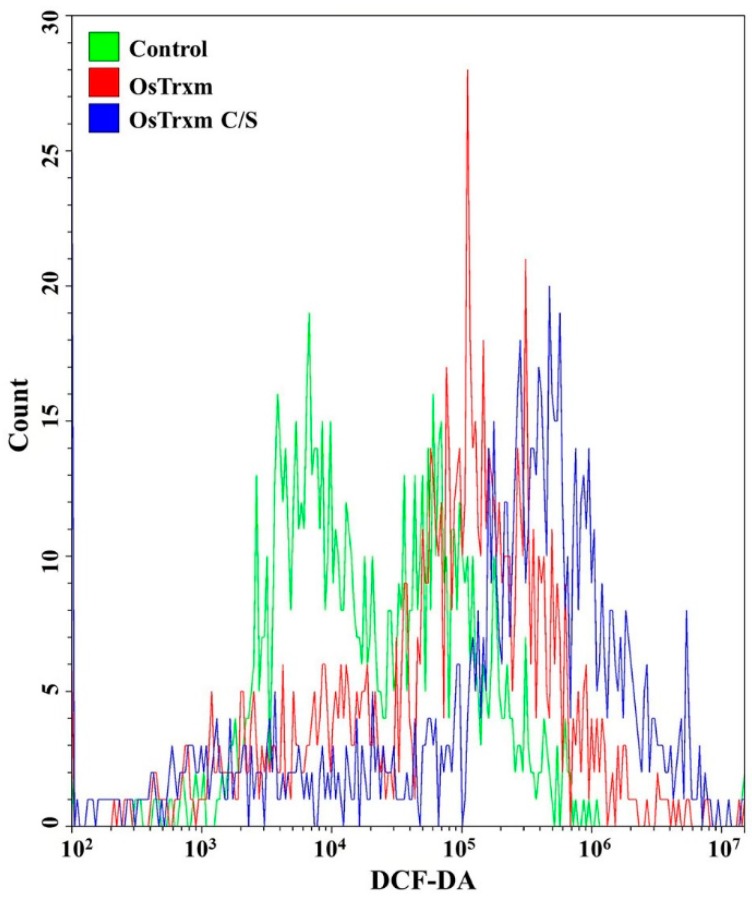
The ROS level in *Aspergillus flavus* was analyzed using FACS with DCF-DA staining after OsTrxm and OsTrxm C/S mutant protein (64 μg/mL and 7 μg/mL, respectively) treatment.

DCF-DA is converted to the fluorescent DCF by oxidative stress, revealing the cellular production of ROS. Figure 4 shows intracellular (A) and extracellular (B) ROS generation by OsTrxm variant proteins. No cells emitted green fluorescence in the presence of melittin, which exerts its antifungal action via pore formation on fungal membrane (Figure 4A). Intracellular green fluorescence was detected in *C. albicans* cells treated with OsTrxm proteins; the number of green fluorescent cells in the presence of OsTrxm C/S was more than that in the presence of OsTrxm (Figure 4A). To investigate extracellular effect of proteins, DCF-DA was added in cell culture medium after 12 h incubation with protein samples (Figure 4B). The two OsTrxm proteins-treated cell culture media and the intracellular part of *C. albicans* cells emitted high green fluorescence under fluorescence imaging system. Although the ROS levels generated were lesser in the presence of OsTrxm than in the OsTrxm C/S-treated cells, OsTrxm stimulated more ROS production than melittin. To confirm the above results, flow cytometry analysis was carried out in *A. flavus* conidia (Figure 5). The right shift observed in case of OsTrxm C/S-treated cells was more than that seen with OsTrxm-treated cells, which is consistent with a better antifungal activity of OsTrxm C/S. The results suggested that OsTrxm proteins exhibit antifungal action via ROS generation.

**Figure 6:**
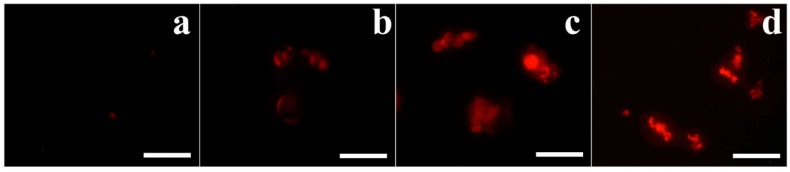
Mitochondrial superoxide (SOX) generation of OsTrxm proteins in *A. flavus* cells. Cells exposed with proteins at IC_50_ concentration for 8 h were stained with MitoSOX Red and observed by using fluorescence microscope. (**a**): control; (**b**): histatin 5; (**c**): OsTrxm; (**d**): OsTrxm C/S. Scale bar: 10 μm.

Furthermore, to investigate intracellular dysfunction of OsTrxm proteins, mitochondrial SOX was detected by MitoSOX Red, a selective fluoroprobe of mitochondrial SOX, after 8 h incubation of proteins in *A. flavus* conidia. Mitochondria in living organisms regulate the excessive ROS production and cell death. Figure 6 shows a generation of mitochondrial SOX in the presence of histatin 5 (b), OsTrxm (c), and OsTrxm C/S proteins (d). Mitochodrial SOX production of OsTrxm proteins was higher than histatin 5. We suggest that antifungal activity of OsTrxm proteins may be due to mitochondrial SOX production in direct action or inhibition of scavenging ability of mitochondria for continuously produced intracellular SOX in indirect action.

### 3.4. Morphological Alterations Caused by OsTrxm Proteins 

**Figure 7:**
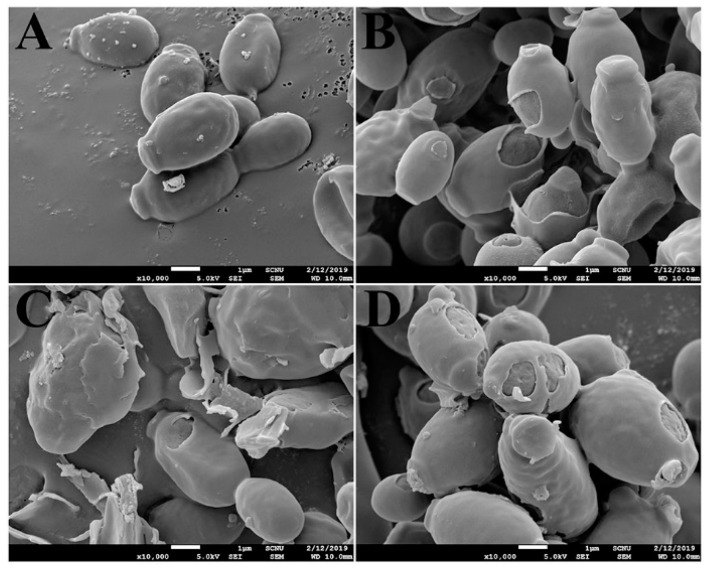
Morphological alterations of *C. albicans* with OsTrxm proteins under SEM. (**A**) control; (**B**) melittin; (**C**) OsTrxm; (**D**) OsTrxm C/S mutant. Scale bar: 1 μm.

Morphological changes of *C. albicans* cells were observed in the presence of melittin, OsTrxm, or OsTrxm C/S by using SEM. After 4 h of incubation, the surface of melittin-treated cells was altered; irregular-sized holes were visible. Similar morphological changes were observed with OsTrxm-treated cells as well (Figure 7). However, *C. albicans* cells treated with OsTrxm proteins exhibited a swollen morphology. These morphological alterations suggest that OsTrxm may exhibit apoptotic action.

## 4. Conclusions

In summary, OsTrxm, a rice protein, was identified as having a novel antifungal function and its antifungal activity can be enhanced by the substitution of amino acids at critical sites. It was found to exhibit a potent antifungal action via generation of mitochondrial ROS and this protein may play an important role in defense system of the rice plant. This may have potential as a lead compound for the development of antifungal agrochemicals or therapeutics.

## Figures and Tables

**Table 1:** Antifungal activity of recombinant OsTrxm proteins against filamentous fungi and yeasts.

Fungal Strains	IC_50_ (μg/mL)	
	Wild	C/S
**Filamentous fungi**		
*Aspergillus flavus*	28	<3.5
*Aspergillus fumigatus*	28	<3.5
*Fusarium moniliforme*	14	3.5
*Fusarium solani*	28	<3.5
*Penicillium verrucosum*	28	3.5
*Phytophthora nicotianae*	14	<3.5
*Trichoderma harzianum*	7	3.5
*Trichoderma viride*	7	3.5
**Yeast**		
*Candida albicans*	14	<3.5
Drug-resistant *C. albicans*	14	<3.5
*Candida catenulate*	28	<3.5
*Candida tropicalis*	28	<3.5
